# Gold standard, multi-genre dataset for named entity recognition and linking

**DOI:** 10.1038/s41597-025-05274-4

**Published:** 2025-06-13

**Authors:** Szymon Olewniczak, Julian Szymański

**Affiliations:** Department of Computer Architecture, Faculty of Electronics, Telecommunications and Informatics, Gdańsk University of Technology, Gdańsk, 80-233 Poland

**Keywords:** Information technology, Software

## Abstract

In our study, we introduce a new dataset designed for the evaluation of entity-linking systems. Entity Linking (EL) involves identifying specific segments in a text so-called mentions and linking them to relevant entries in an external Knowledge Base (KB). EL is a challenging task with numerous complexities, making it vital to have access to high-quality data for testing. Our dataset is unique in that it encompasses texts from various domains, contrasting with the common focus on single domains, such as newspaper news, in most current datasets. Furthermore, we have annotated each identified text segment with its corresponding entity type, enhancing the dataset’s usefulness and reliability. This dataset employs Wikipedia as its Knowledge Base, which is the prevalent choice for general domain entity linking systems. The dataset is available to download from 10.34808/f3q9-9k64.

## Background & Summary

Entity linking (EL) is an important task in Natural Language Processing. The main focus in EL is methods and algorithms that extract important concepts from raw text input and link them with corresponding entities in the selected Knowledge Base. There are two main categories of EL systems: general and domain-specific. The first category focuses on linking to general domain knowledge bases such as Wikipedia, Wikidata, DBPedia, Yago, or Freebase. Another uses some domain-specific knowledge, for example, medical. In this work, we focus on the systems in the first category.

The Entity Linking process consists of two important steps. The first is called Mention Detection (MD). In this stage, we find phrases in our input text that we consider important to our algorithm. Formally, we can define a function that takes the raw text *T* as input and returns the set *M*^*n*^ = {*m*_1_, …, *m*_*n*_} of detected mentions:

1 $$MD:T\to {M}^{n},$$

Usually, we assume that the detected mentions are continuous fragments of the input text. In this case, we can define *m*_*i*_ = (*s*_*i*_, *l*_*i*_) where *s*_*i*_ is the starting character of the mention and *l*_*i*_ is its length.

The second stage of the EL pipeline is called Entity Disambiguation (ED). Here, for every detected mention, we find a corresponding entity in our KB or (in some setups) decide that there is no matching entity (which is sometimes called *NIL* detection). Formally, this step can be defined as

2 $$ED:{(M,C)}^{n}\to {(E\cup NIL)}^{n}.$$

where *C* is the context of the disambiguated mention and E is a set of entities in KB.

Many general domain EL systems use Wikipedia articles as training/testing datasets. However, since the EL algorithms are meant to work on a wide variety of input texts, we are interested in their performance on the texts outside the Wikipedia with its specific encyclopedic style. To tackle this requirement„ many evaluation datasets for the EL problem were proposed. Now, we will present some of the most prominent examples.

The first widely known evaluation dataset for the EL problem was MSNBC^1^. To create the dataset, the top two stories from each of the 10 MSNBC news categories were taken and processed by the EL system proposed by the authors of the article. To create the gold standard, the wrongly identified mentions were removed and incorrectly disambiguated links were fixed.

In the next year, AQUAINT^2^ was released. It consists of 50 news articles (250-300 words long) with links to Wikipedia created in a crowd-sourcing effort. The dataset was intended to mimic the links created by Wikipedia contributors, so the references with which to link were selected for their subjective usefulness to the reader.

In 2011 two other interesting datasets were proposed. The first was ACE2004, created to evaluate the GLOW system^3^. It was based on the mentions selected in a subset of ACE 2004 Multilingual Training Corpus^4^ that were linked to the corresponding Wikipedia articles.

The second was AIDA/CoNLL^5^, which was created by adding target links to the mentions of the CoNLL 2003 dataset^6^. This dataset is much larger than previous ones (1,393 texts with 34,929 mentions) and additionally is distributed with pre-selected training/validation/testing split, which allows us to use it not only for testing but also for training EL systems.

The last interesting dataset that we want to present in this summary is MultiNERD^7^. MultiNERD was created as a silver standard dataset, which makes it a little less precise; however, it is much larger (253,455 links). Additionally, every mention in the dataset contains the assigned type, which makes it much more useful.

There are several problems with current gold standard evaluation datasets. First, there is no general acceptance of which entities should be considered by EL systems^8^. When we link to Wikipedia, it is clear that we do not want to link with every single entity (which includes articles on topics such as “the” article), but the exact subset is hard to define. Another somehow related question is which mentions should be detected during the MD phrase. One of the ideas to define this phrase more precisely is to specify the set of entity classes that we want to consider^9^, but there are still some more questions to answer. Finally, gold-standard datasets usually cover some narrow domain of possible text inputs, such as news articles that may give biased results.

To address some of these issues, we propose a new gold standard dataset for EL, called Elgold. The most important features of Elgold are the following:

- Covers a diverse set of raw texts.
- MD stage is precisely defined using annotation guidelines and a pre-defined set of named entity classes. It can be used both for NER and NED tasks.
- All mentions are assigned to the proper class. Mentions that do not have corresponding Wikipedia articles are left as NIL links.
- Each linked entity was checked by at least three people.
- It is based on the last version of Wikipedia (2023-11-21).

Compared to other datasets in the domain, Elgold comes with some unique characteristics:

- The raw texts present a wide variety of topics and styles, which is uncommon in other popular datasets.
- Every mention is assigned to a proper class, similar to MultiNERD. But unlike in MultiNERD all were carefully checked by humans.
- Although Elogld is smaller than AIDA/CoNLL, it is still much larger than other gold standard datasets such as MSNBC, AQUAINT, and ACE2004.

Table 1 summarizes the differences between Elgold and other popular datasets.

**Table 1:** Comparison of EL datasets. For comparison we use revised versions of MSNBC, AQUAINT and ACE2004 from^25^.

Dataset	Source	Texts	Mentions	Links	NER classes	Creation method
MSNBC	Selection of MSNBC news.	20	739	656	n/a	Manual verficiation of algorithm results.
AQUAINT	Subset of AQUAINT text corpus.	50	727	727	n/a	Manual verficiation of algorithm results.
ACE2004	Subset of ACE 2004 dataset.	57	306	257	n/a^*^	Links on top of ACE 2004 dataset.
AIDA/CoNLL	CoNLL 2003 dataset.	1393	34,929	31,816	n/a^†^	Links on top of CoNLL 2003 dataset.
MultiNERD EN	Wikipedia, WikiNews	n/a^‡^	253,455	253,455	15^*§*^	Silver data standard.
Elogld	various	276	3,559	3,106	14^*§*^	Separate MD and EL stages.

^*^Original ACE 2004 dataset contained 7 NER classes (with additional subtypes)^4^ but they where removed by the EL dataset authors^3^.

^†^Original AIDA/CoNLL dataset contained 4 NER classes: PER, LOC, ORG, MISC^6^ but they where removed by the EL dataset authors^5^.

^‡^The dataset is distributed as a set of sentences.

^*§*^Modified OntoNotes 5.0 schema.

Similarly to MSNBC, AQUAINT, and ACE2004 Elgold is primary developed as test dataset but can be also used as a high quality dataset in zero/few-shot setup in LLM based NER/EL systems such as:^10–12^ among others.

We believe that the Elgold dataset contributes to the EL domain and may shed new light on some of the important aspects of the field.

## Methods

Gathering data to create the gold standard EL dataset is hard. This is mainly due to the lack of a precise definition of the task. The first studies in the field followed Wikipedia’s Editing policy (Wikipedia: Editing policy), which recommended linking only to pages that may interest the reader^2,13^. However, this led to many inconsistencies between different annotators, and it quickly became obvious that we needed something more precise.

The creation of a formal definition of EL is very challenging and probably could never be achieved, since the natural language itself cannot be precisely defined. However, over the years, there have been some attempts in the field to somehow unify the work of different annotators^8,9^. This is usually achieved by creating annotation guidelines.

### Guidelines for annotators

When creating annotation guidelines, we must consider several important aspects. Firstly, we must decide on the technical aspects of annotations. This includes three main points:

1. Do we allow annotations to overlap or any given text fragment can point to at least one entity? For example, the text fragment: “Gdańsk University of Technology” probably should be linked to the Wikipedia article: “Gdańsk University of Technology” but when we allow overlapping annotations, we can also link the name of the city “Gdańsk” to the corresponding article.
2. Should the detected mentions be continuous fragments of text or do we allow holes between mention fragments? For example in the sentence: “Castor and his twin-brother Pollux helped the Romans at the Battle of Regillus.” we may want to link the mention “Castor and Pollux” to the corresponding article, without the “his twin-brother” phrase.
3. Do we allow character-level mentions or consider only word-level ones? Charter-level mentions allow the links to start and end in the middle of a word; for example, we can link the word “foot” to the phrase “football”. However, word-level mentions can start only with the beginning of the word and end with the word ending.

When we decide on the technical aspects of links, we must face another problem - what kind of entities do we want to detect. There are two main general approaches to this challenge. The first is called KG-dependent and the other is KG-independent. In the KG-dependent approach, we assume that all mentions detected by our system must be linked to some entity in the KG (we do not allow NIL links)^2,13–15^. In other words, the KG influences the MD stage by defining what a valid mention is and what is not. The mention detection stage in KG-dependent EL systems is usually based on generating the surface forms of entities in KG and then detecting them in the input text.

In the KG-independent approach, we treat mention detection and entity disambiguation as two separate tasks. Usually, authors of this kind of systems focus only on the ED stage, delegating the MD stage to external tools (usually NER algorithms)^16,17^. This kind of systems should include some form of NIL detection to work properly because we have no guarantee that all mentions detected by NER have their corresponding entity in our KG.

In the context of preparing gold standard datasets for EL, annotators using the KG-dependent approach are asked to find the mentions in text that have their corresponding entity in KG and link them. On the other hand, annotators using the KG-independent approach first detect the relevant mentions (or use the input with mentions already detected), and later decide if they can link them to KG or left as NIL-links.

Another important question we must answer is what kind of mentions do we want to detect during annotation. This usually boils down to the decision do we want to link common concepts or only focus on named entities? Common concepts can sometimes be helpful, but it is very difficult to define which common concepts should be considered important to maintain consistency between different annotators. The named entities are much more concrete, but still require some clarification.

Although we usually understand intuitively what a named entity is and what is not, there is a lot of confusion in some borderline examples. When talking about named entities, we usually refer to the definition provided by S. Kripke^18^. In his work, he defined the so-called rigid designator. Something is a rigid designator if “in every possible world it designates the same object”. In practice it means that we consider as named entities proper names and certain natural kind terms like biological taxonomy and natural substances.

Selecting named entities, based only on a rigid designator, can still be problematic. To make this procedure more concrete, we usually define so-called NER classes which help to decide which mentions should be selected from the raw text. NER classes are defined once for the entire annotation process in the NER classes scheme. There are currently two most popular NER class schemes. One defined by CoNLL 2003^6^ contains four classes: person, organization, location, and miscellaneous. Another popular NER class scheme is OntoNotes 5.0^19^. It defines 18 NER classes: cardinal, date, event, fac, geopolitical entity, language, law, location, money, nationalities or religious or political groups, ordinal, organization, percent, person, product, quantity, time, and work of art. Strictly speaking, not always all entities of interest defined in the NER classes scheme are named entities in the sense of rigid designator and the other way round: not all possible named entities are covered by the NER classes scheme. However, the good-classes scheme with descriptions and examples provides a common base for all annotators.

To sum up, good annotation guidelines should at least define the technical aspects of annotations, the kind of mentions detection procedure (KG-dependent, KG-independent), the type of mentions we want to select (named entities, common names), and if we decide on named entities, the classes of interest.

### Overview of the dataset creation

**Fig. 1:**
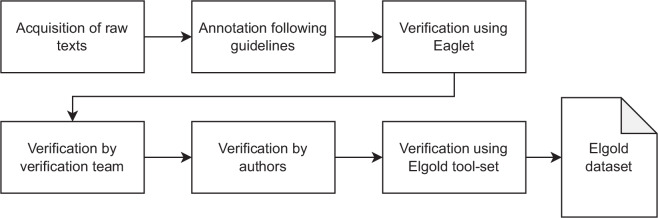
The overview of the dataset cration process.

Our dataset creation procedure consists of several stages. Some of them involve human participants, and some are performed by validation tools. The overview of our entire dataset preparation pipeline is presented in Fig. 1. The big problem in creating EL gold standards is ensuring that the results are consistent between different annotators. We achieved this by providing the annotation guidelines and performing four verification steps, two of which were automatic and two manual. We explain each of the stages in detail in the following sections.

Popular technique of assuring annotations correctness is inter annotators agreement. In this technique, we give the same text for annotation to several people and then select only the annotations that are selected by the majority of annotators. However, this technique may lead to the omission of harder entities that may not be obvious to humans. In other words, it maximizes the precision of the annotations, but may lower the recall. The inter-annotation agreement also requires significantly more resources since one text must be annotated by several people. In creating the Elgold dataset, we applied an alternative technique that is based on multistage validation. This gives more freedom to verifiers and may help them extract more difficult entities from the texts.

### Elgold annotation guidelines

In the following, we present the annotation guidelines that were presented to all text annotators before they started their work. Although no guidelines are complete, they provide a solid foundation and cover the most important aspects of the annotation procedure.

1. All detected mentions should be non-overlapping, continuous, and word-level.
2. All detected mentions should be named entities with a specific class assigned. For maximum utility, we decided to base on the OntoNotes 5.0 schema^19^, which is the industry standard, but we modified the original classes set a little bit to better fit the EL task. The complete list of NER classes used in our dataset is presented in Table 2. More details about the differences between our class set and the OntoNotes schema are described in the next section.

**Table 2:** NER classes used in the dataset.

Our Class	Description
EVENT	Named hurricanes, battles, wars, sports events, etc.
FAC	Buildings, airports, highways, bridges, etc.
GPE	Countries, cities, states
LANGUAGE	Any named language
LAW	Named documents made into laws.
LOC	Non-GPE locations, mountain ranges, bodies of water
NORP	Nationalities or religious or political groups
ORG	Companies, agencies, institutions, etc.
PERSON	People, including fictional
PRODUCT	Objects, vehicles, foods, etc. (not services)
WORK_OF_ART	Titles of books, songs, etc.
DISEASE	Names of diseases
SUBSTANCE	Natural substances
SPECIE	Species names of animals, plants, viruses, etc.
3. We use the KG-independent approach. The annotators first mark the proper mentions and then try to link them to KG if possible.
4. We use Wikipedia articles as our KG. All detected mentions should point to the corresponding Wikipedia articles if exist. We do not allow redirects, disambiguation pages, or fragments of articles to be considered entities. If the article describing the mention does not exist, we left it as NIL-link.
5. When two possible mentions overlap, we should select the longer one, which is usually more specific. For example, when the article contains the phrase United States Congress, we should link it to an article about the United States Congress and not link the phrase “United States”.
6. We should always link to the most specific entity possible. For example, when the article refers to the United States Congress, and we know for the context that this is the 118th US Congress we should link to this more specific article.
7. We should respect the metonymy. So, for example, the phrase “Kyiv” should be linked to “Government of Ukraine” when it refers to the government, not the city itself.

### Elgold NER Classes

Similarly to some other work^7,20^, we took the OntoNotes 5.0 classes scheme and modified it to better fit the EL task and our specific dataset. Firstly, we removed 7 classes: CARDINAL, DATE, MONEY, NUM, ORDINAL, PERCENT, QUANTITY, and TIME which were intended to link to specific numerical values or points in time that do not make sense in the context of EL. Then we added 3 additional classes: DISEASE, SUBSTANCE, and SPECIE which can also be considered as well-defined named entity classes and are very important in some of our raw text categories. The complete list of NER classes in our dataset is provided in Table 2. The compression between our NER classes OntoNotes scheme and Tedeschi and Navigli^7^ is presented in Table 3.

**Table 3:** Comparison between our NER Classes, OntoNotes and Tedeschi and Navigli.

Our Class	OntoNotes Class	Tedeschi and Navigli^7^
EVENT	EVENT	EVE, TIME
FAC	FAC	LOC
GPE	GPE	LOC
LANGUAGE	LANGUAGE	MEDIA
LAW	LAW	—
LOC	LOC	LOC
NORP	NORP	ORG
ORG	ORG	ORG
PERSON	PERSON	PER, MYTH*
PRODUCT	PRODUCT	FOOD, INST, VEHI
WORK_OF_ART	WORK_OF_ART	MEDIA
DISEASE	—	DIS
SUBSTANCE	—	—
SPECIE	—	ANIM, BIO, PLANT
—	—	CEL

Some of the entities in MYTH class may not be classified as PERSON in our scheme.

### Acquisition of raw texts

The first step in creating our dataset was the acquisition of raw texts, which formed the basis for the annotation. We have several important considerations when selecting the raw texts for future processing:

1. We wanted to collect recent texts that cover the latest trends and events.
2. We wanted texts from different domains, with different language styles and with different topics covered.
3. We wanted texts of various lengths.

Finally, we decided to collect the texts into seven main categories: “News”, “Job offers”, “Movie reviews”, “Automotive blogs”, “Amazon product reviews”, “Scientific papers abstracts”, and “Historic blogs”. The Scientific Papers category was additionally divided into five subcategories: “Biomedicine”, “Life Sciences”, “Mathematics”, “Medicine & Public Health”, and “Science, Humanities and Social Sciences, multidisciplinary”.

The texts’ categories were selected by authors and collaborators during the brain-storming and were kind of the trade-off between ease of acquisition and diversity. Our subsequent research (described in the Technical Validation “Section”) actually showed an increase in textual diversity relative to similar datasets.

The raw texts were collected from publicly available Internet sources by the group of 14 participants. Each category has 2-3 participants assigned. After this step, we have a repository of approximately 100 texts for each category (and subcategory in the case of “Scientific papers abstracts”). Table 4 summarizes the basic statistics of the raw texts. For the rest of the paper, we will treat the subcategories of “Scientific papers abstracts” as one standalone category.

**Table 4:** The statistics of the raw text collected.

Id	Category name	Count	Min	Max	Avg	Std
1	News	99	98	784	288	112
2	Job offers	90	88	1176	397	221
3	Movie reviews	100	68	234	138	41
4	Automotive blogs	100	98	464	188	71
5	Amazon product reviews	100	108	207	156	20
6	Scientific papers abstracts	779	10	501	156	83
6a	Biomedicine	180	21	501	154	85
6b	Life Sciences	167	10	325	167	80
6c	Mathematics	140	14	305	117	63
6d	Medicine & Public Health	96	14	355	96	82
6e	Science, Humanities, ...	196	51	414	207	60
8	Historic blogs	50	93	378	202	55
	Total	1318	10	1176	185	116

### Annotation following guidelines

The next step of our data acquisition was to annotate the raw texts following the guidelines. This step involved 31 participants. During this step, participants annotated the named entities found in the raw texts and linked them to the corresponding Wikipedia articles.

The annotators were split into 3 groups, counting 15, 5 and 11 participants, respectively. Each participant worked for 1,5 hours on the task. Each group was supervised by one of the dataset authors, so in case of any doubts, the annotator can ask for clarification.

To facilitate the annotation process, we have created a dedicated web tool ligilo. The ligilo can be downloaded from GitHub: https://github.com/solewniczak/ligilo. The tool offers two important features. Firstly, it is integrated with the spaCy (https://spacy.io/) NER module, which can speed up the process of mention detection a bit. The spaCy NER is based on OntoNotes 5.0 schema, so it does not include all the classes that we use in our dataset, but it still provides a good starting point. The second important feature of ligilo is the integration with the Wikipedia API (https://en.wikipedia.org/w/api.php) which allowed the participants to search for the corresponding articles, similar to Wikipedia’s visual editor (https://en.wikipedia.org/wiki/Wikipedia:VisualEditor).

**Fig. 2:**
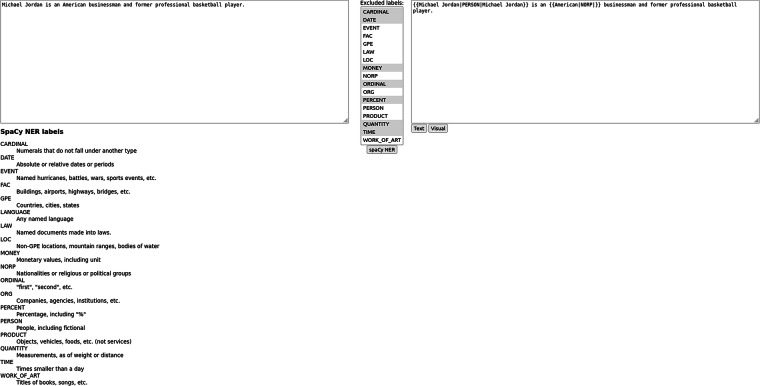
Ligilo text editor.

**Fig. 3:**
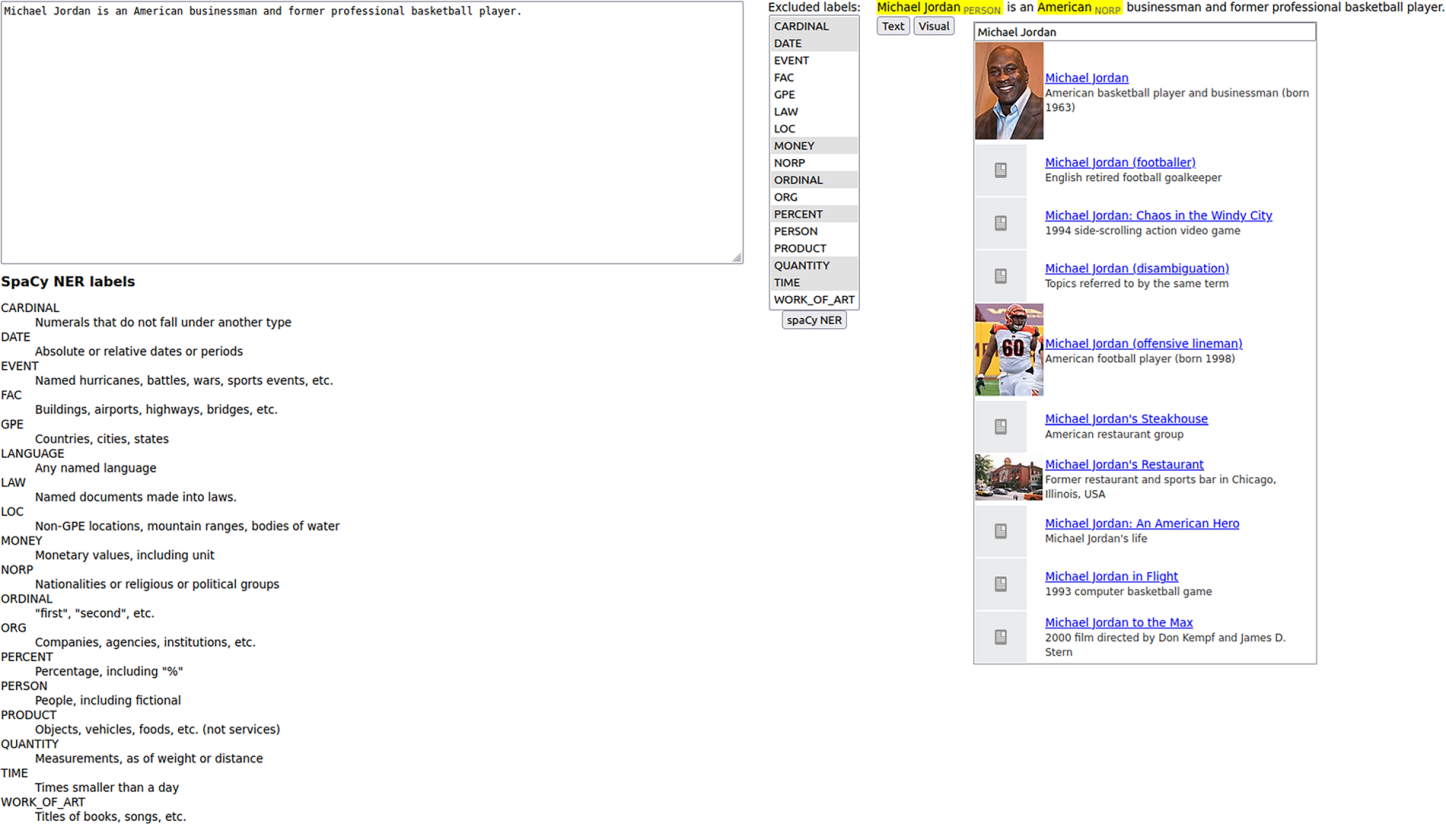
Ligilo visual editor.

The ligilo interface is split into two parts (Fig. 2). The left part allows the annotator to insert the raw text input, and the right part contains the annotated output. Between the parts, there is a button that allows the spaCy NER (with selected classes) to be run on the raw text input. The output can be presented in two views. The textual view (Fig. 2) allows one to insert, remove or edit the boundaries of the mentions, and specify the mention classes. The visual view (Fig. 3) is integrated with the Wikipedia API and allows one to find the correct entity for the mention. The details of the output format are described in the Data Records section.

**Fig. 4:**
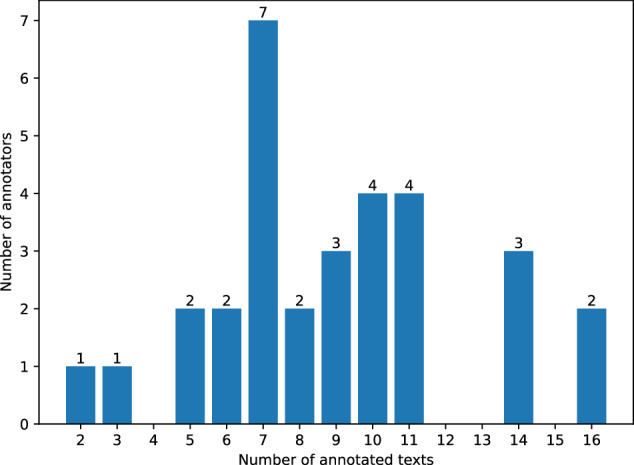
Number of annotators who annotated specific number of texts.

Before starting the annotation work, the annotators were instructed to focus on correctness, rather than speed. So, during the 1.5-hour session, they annotated as many texts as they managed to. Each participant started the annotation from the first available text (not processed by anyone else) from the 1st category, then moved to the 2nd category, and so on. After processing the text from the last category, they started from the beginning. Every raw text was processed only once by one annotator. Figure 4 presents how many texts were annotated by individual annotators. We can observe that most of the participants annotated between 5 and 11 texts.

After this step, we have collected 276 annotated texts for all 11 categories with 3404 annotations. Table 5 presents the textual statistics of the subset of raw text that was annotated. As we may see, the later categories have a smaller number of texts than earlier because not all participants manage to complete the whole annotation cycle.

**Table 5:** The statistics of the collected annotated texts.

Id	Count	Min	Max	Avg	Std
1	37	176	479	293	82
2	34	108	1176	513	259
3	37	68	227	137	46
4	34	103	192	141	22
5	34	117	192	155	18
6a	27	36	279	167	73
6b	21	87	350	208	62
6c	16	58	239	118	52
6d	14	17	271	133	93
6e	9	142	321	196	54
8	13	128	378	217	74
Total	276	17	1176	220	161

### Verification using Eaglet

Because linking text with entities is a hard task, human annotators often make mistakes. To mitigate this issue, we implemented a series of verification steps to create a high-quality dataset. Firstly, we tried the Eaglet^9^ tool, which aims at automatic verification of the gold standard EL dataset.

Eaglet automatically detects 8 types of annotation errors:

1. Long Description Error (LDE) – checks for mentions that contain more than just a surface form of an entity.
2. Positioning Error (PE) – checks for mentions that begin or end in the middle of a word.
3. Overlapping Error (OE) – check for overlapping mentions.
4. Combined Marking (CM) – checks for consecutive mentions, which usually can be combined into a single more precise mention.
5. Outdated URI (OU) – checks for non-existing links’ targets.
6. Disambiguation URI (DU) – checks if the link points to a disambiguation page.
7. Invalid URI (IU) – check for badly formatted URIs.

From the perspective of our annotation guidelines, all these annotation errors make sense, except the OE which cannot occur in our dataset format. The number of annotation errors found by Eaglet and fixed by us is presented in Table 6.

**Table 6:** The errors detected by Eaglet and finally fixed.

	found	% found	fixed	% fixed
LDE	3	0.09%	2	0.06%
PE	23	0.68%	7	0.21%
OE	0	0.00%	0	0.00%
CM	61	1.79%	34	1.00%
OU	0	0.00%	0	0.00%
DU	0	0.00%	0	0.00%
IU	0	0.00%	0	0.00%
IM	8	0.24%	3	0.09%

Total number of annotations: 3404.

### Verification by the verification team

Unfortunately, after examining the Eaglet-fixed dataset, we were still not satisfied with the quality of the annotations. There were still many links that were wrongly placed or pointed to incorrect pages. To make our dataset more reliable, we decided to implement an additional manual verification step.

After the preliminary examination, we identified 8 types of errors that were common in the documents:

1. Long – mention was too long.
2. Short – mention was too short.
3. Removal – mention cannot be assigned to any of NER classes and should be removed.
4. Addition – in the raw text exists a mention that can be marked or not all occurrences of mention were added.
5. Class – mention was added to the wrong NER class
6. Syntax – syntax error in mention marking
7. Link – mention points to wrong Wikipedia page
8. ’s – saxon genitive should not be included in mention

We once again hired a group of 5 participants (we called them editors) to check the dataset and apply the required corrections. Each of the editors received an equal portion of the texts from each category.

The work of the correction team differs from the annotators in several important aspects:

- Each editor saw the work of different annotators, so they could confront their own opinions with the other point of view.
- They were not limited in time, so they could calmly consider any uncertainties.
- The editors were in constant contact with each other and with the authors of the dataset, so any doubts could be discussed.

The correction procedure proved to be very fruitful. Almost 25% of the links were corrected in some aspect. Table 7 provides the exact number of corrections made in each error class.

**Table 7:** The errors fixed by editors.

	fixed	% fixed
Long	136	4.00%
Short	53	1.56%
Removal	64	1.88%
Addition	249	7.31%
Class	176	5.17%
Syntax	12	0.35%
Link	100	2.49%
’s	45	1.32%
Total	835	24.53%

Total number of annotations: 3404.

### Verification by the authors

After the correction team finished its work, the authors again reviewed all the texts to check for additional inconsistencies or errors. After that step, our verified dataset consists of 3559 mentions, so the number of mentions has grown compared to raw annotations.

### Verification using the Elogld toolset

Finally, we applied some automatic verification and correction steps for our dataset. They were performed using our dedicated Elogld toolset and standard Unix text processing utilities:

1. Fixing misspellings in NER class names.

2. Replacing some Unicode charters which have close ASCII equivalent. The mappings used are available in Table 8.

**Table 8:** Mappings of selected Unicode chars to their close ASCII equivalents.

Character	UTF-16 encoding	Name	Replace with
’	0 × 2019	Right Single Quotation Mar	’
	0 × 2009	Thin Space	Space
”	0 × 201C	Left Double Quotation Mark	”
”	0 × 201D	Right Double Quotation Mark	”
–	0 × 2013	En Dash	–
—	0 × 2014	Em Dash	–
	0 × 2212	Minus Sign	–
‘	0 × 2018	Left Single Quotation Mark	’

replace-chars --unicode-escape --delete "\u2022"

"\u2019\u2009\u201C\u201D\u2013\u2014\u2212\u2018""'\u0020\"\"—'"

3. Targets normalization. We normalized all targets using the Wikipedia API.

fix-targets --normalize.

4. Removing non-existing targets. We searched for links to non-existing pages and fixed or removed them.


fix-targets --remove-non-existent --interactive


5. Resolving redirects. We checked for links that link to Wikipedia’s redirect pages and changed them to final destinations.


fix-targets --redirect --interactive


6. Removing Saxon genitive and quotes from the mentions. We used grep tool to search for Saxon genitives and quotes and removed them from the mentions. Additionally, we removed the honorific “Mr” and “Mrs” from the mentions, but kept more specific ones: “King”, “Prince”, “Pope”, “Sant”, “St.”, and “Baron”.

During this step, some additional manual fixes were also made where we found it necessary. The created dataset contains valid links to Wikipedia as of 2023-11-21.

## Data Records

The dataset is available at Bridge of Knowledge^21^, with this section being the primary source of information on the availability and content of the data being described.

The dataset is distributed as a set of textual files. Each file contains an annotated article. File names follows following convention: {category id}_{serial number}.txt. For example, a file named: 6c_10.txt means the 10th article from the “Scientific papers - Mathematics” category. Sometimes annotated articles contain holes in the sequence numbers. This happens because during the annotation step some participants “locked” the articles that they did not manage to annotate.

The annotations are embedded directly in the articles and use a syntax similar to Wikipedia templates:

{ { mention content ∣ entity class ∣ target } }

For example:

Best Actor due to his work on { { Cracker ∣ ORG ∣ Cracker (British TV series) } }

The mention content and entity class cannot be empty. The target can be empty when the entity does not have the corresponding Wikipedia article but the third pipeline charter must be present. For example:

He also appeared as the villain { { Valentin Zukovsky ∣ PERSON ∣ } }

The charters {and} are used only to mark mentions and are never used as raw text content. This allows us to match all the entities in the dataset using a simple regular expression:

{ { [^ { }]* } }

**Fig. 5:**
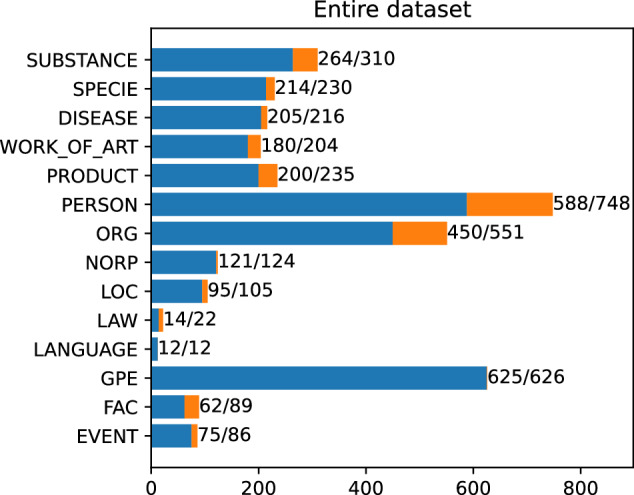
The number of entities in each of NER classes. The first number shows the quantity of linked entities, the second all marked mentions.

**Fig. 6:**
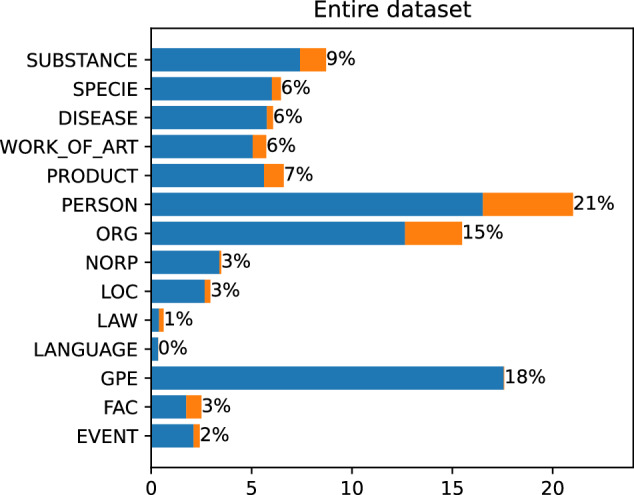
Number of links in each of NER classes. Percentage shares.

The final dataset consists of 3559 marked mentions. 3106 mentions have their corresponding articles and are linked to Wikipedia. The remaining 453 are valid named entities, but do not have their corresponding Wikipedia articles. Each of the mentions is assigned to one of 14 NER classes (see Table 2). Figures 5 and 6 present the distribution of NER classes throughout the dataset.

**Fig. 7:**
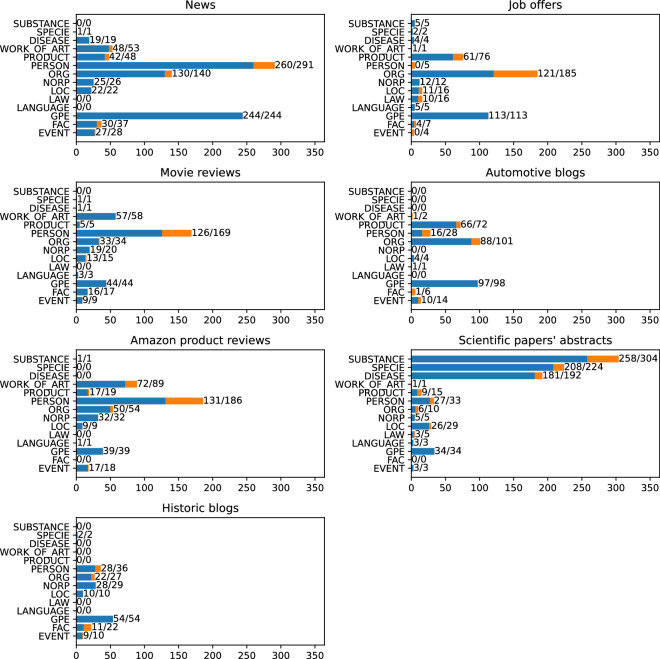
Number of links in each of NER classes, split into separate categories. The first number shows the quantity of linked entities, the second all marked mentions.

**Fig. 8:**
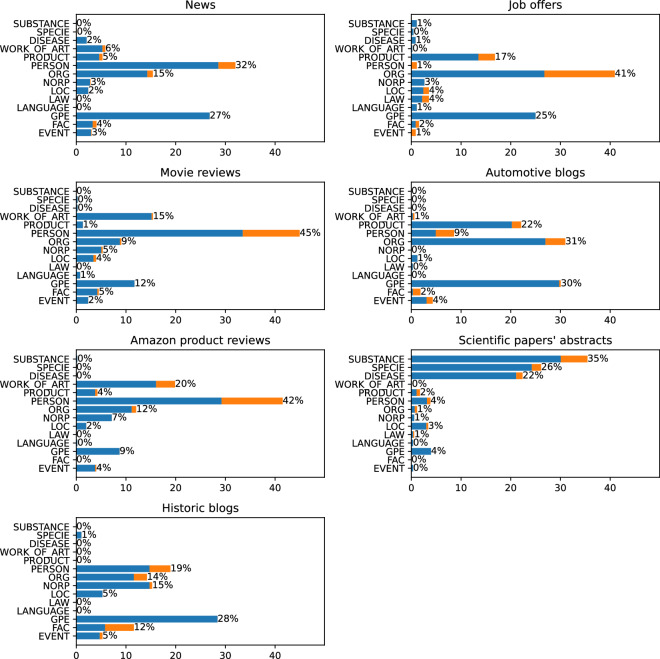
Number of links in each of NER classes, split into separate categories. Percentage shares.

To get a better picture of the data, we checked the distribution of the NER classes for each of the text categories. Figure 7 shows the absolute number of links in each of the classes, while Fig. 8 shows the percentage. The results show that different category of articles have a diversified class distribution.

In the News category, we may observe that the PERSON is the most popular class, with the strictly following GPE. This reflects the main topic of such texts that consider persons who do something in specific places.

When we look at the “Job offers”, we can observe a different trend. Here we have a lot of links that concern some organizations as can be expected in this category of raw texts. The GPEs are mainly the places where the work should be performed. PRODUCTs are usually related to the candidate’s expected skills.

The next category in our dataset is Movie reviews. Here, the dominant NER class is PERSON, followed by WORK_OF_ART. Here, the links mainly involve actors, directors, and fictional characters on the one hand and titles of the movies on the other.

When we move to Automotive blogs, we can observe that the dominant class is ORG, strictly followed by GPE and PRODUCT. This is because the texts are focused on cars and their manufacturers.

In the Amazon product reviews category, we see surprisingly few links in the PRODUCT class. However, we must remember that the WORK_OF_ART category also covers many product types such as books, movies, or music albums. Here, similarly to Movie Reviews, the PERSON class is the most popular, which includes book and music authors, movie directors, and actors.

The next class in our dataset is the Scientific papers’ abstract, which is primarily focused on biology. Here we can observe a very different distribution of NER classes. Three main classes here are SUBSTANCE, SPECIE, and DISEASE which are classes outside the OntoNotes scheme. This clearly shows that the currently most popular annotation scheme may be inadequate for some types of raw text.

Finally, we have the Historic blogs category. In this category, the GPE class is the most popular one, which makes sense since most of the history is, in fact, political history.

## Technical Validation

To test the technical quality of our dataset we need to check two aspects of our dataset. The first is the “mention detection” stage. In our case, MD is defined as detecting named entities from defined classes. The second aspect is Entity Disambiguation, so we want to know if our mentions are correctly linked with Wikipedia articles.

Although there are some EL systems that perform MD and ED jointly (^14,15,22^ among others) they cannot be used reliably in our case. The main reason is that they do not define the “mention detection” stage in terms of annotation rules but are usually fine-tuned on the part of the target dataset and try to learn the rules from the data. It may work on the Elogld dataset, but in our opinion, the results would be less informative.

The alternative for jointly doing MD and ED is to split the process into two separate stages. When we do so, we can check separately how good we are at mention detection and our performance on disambiguation using the so-called linking with gold mentions. This means that we remove links’ targets from the dataset and then disambiguate mentions using the algorithm.

### Measuring texts diversity

One of main contributions of the Elgold dataset is offering more diversified texts base in sense of their topic and style than in currently available datasets in EL domain. We achieved that by selecting raw texts from diverse sources. However, to test our assumptions, we created an objective procedure that allows us to measure the diversity of the EL datasets.

Our diversity testing procedure is based on two main components. The first is the DBPedia Ontology (https://www.dbpedia.org/resources/ontology/), which categorizes Wikipedia articles into hierarchical structures. The second is DBpedia Spotlight^23^ that allows to annotate raw texts with links to DBPedia^24^.

The developed procedure consists of several steps. Firstly, we mapped the DPBedia Ontology categories into our named entity classes. The developed mappings are presented in Table 9. Then we used DBPedia Spotlight to annotate raw texts with the links to DBPedia. The created links are then mapped to the corresponding named entity classes. For each link, we get its base category from the DBPedia and then move up the categories hierarchy until we find one of our mappings category. Some of the links are removed if they do not map to any given category. Finally, the mappings for each raw text are summed across the dataset, creating a histogram of named entities. Based on this kind of data, we can use statistical measurements such as the Gini index to compare the histograms for different datasets.

**Table 9:** Mappings of Elgold classes to DBPedia Ontology.

Elgold class	DBpedia Ontology
EVENT	Event, SportsSeason
FAC	ArchitecturalStructure
GPE	Place/PopulatedPlace
LANGUAGE	Language
LAW	Work/WrittenWork/Law
LOC	Place/NaturalPlace
NORP	EthnicGroup
ORG	Agent/Organisation
PERSON	Species/Eukaryote/Animal/Person, FictionalCharacter, Deity
PRODUCT	Device, MeanOfTransportation, Food
WORK_OF_ART	Work -Work/WrittenWork/Law
DISEASE	Disease
SUBSTANCE	ChemicalSubstance, Biomolecule
SPECIE	Species -Species/Eukaryote/Animal/Person

In the “DBPedia Ontology” column the minus sign means that we exclude the given subcategory from the base category.

**Fig. 9:**
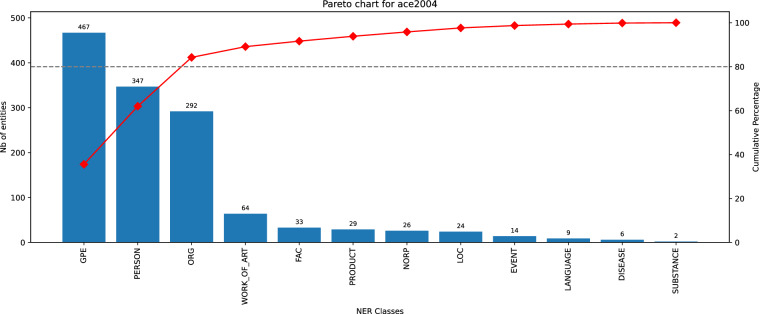
Histogram of entity classes distribution for ACE2004 dataset.

**Fig. 10:**
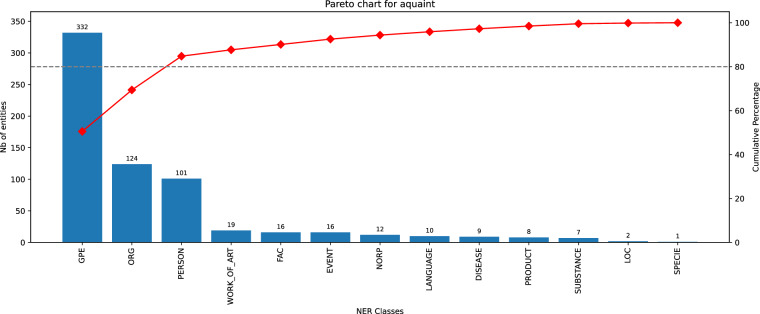
Histogram of entity classes distribution for AQUAINT dataset.

**Fig. 11:**
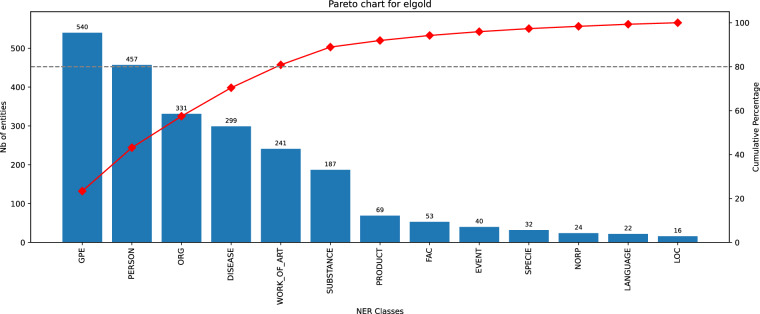
Histogram of entity classes distribution for Elgold dataset.

**Fig. 12:**
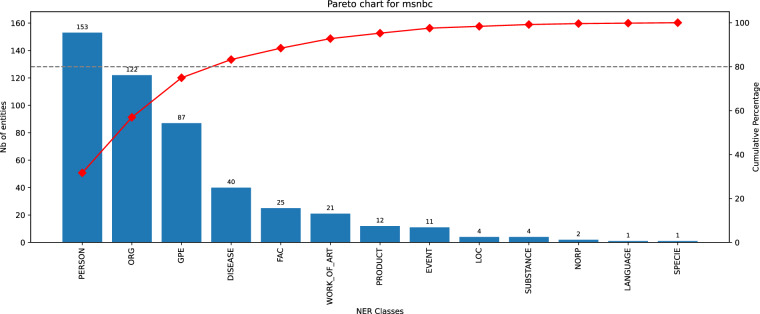
Histogram of entity classes distribution for MSNBC dataset.

We used the described procedures to compare Elgold with MSNBC, AQUAINT and ACE2004 datasets. In the Figures 9, 10, 11, 12 we can see the histograms created for each dataset with the Pareto cumulative line showing the growing percentage contribution of each category to the total. In these figures we can observe that the Elgold dataset is clearly better balanced than the other datasets.

Additionally to visual analysis we can calculate the Gini index for each dataset:

3 $$G=\frac{{\sum }_{i=1\,}^{n}{\sum }_{j=1}^{n}| {x}_{i}-{x}_{j}| }{2n{\sum }_{i=1\,}^{n}{x}_{i}}$$

where *n* is the number of entity classes in dataset histogram, *x*_*i*_ is the number of entities in the given class. Lower Gini index means a more diversified dataset. In our four datasets, we have the following Gini indices:

- ACE2004 - 0.6629
- Elgold - 0.5248
- MSNBC - 0.6425
- Aquaint - 0.7083

We can see that the Gini index for Elgold is much lower than in the other dataset, which suggests its better diversity.

### Mention Detection using SpaCy

In the Elgold dataset annotation guidelines, we decided to use named entities as our mentions. This allows us to validate the results obtained using the NER algorithms. Since our class set used for labeling named entities is based on the popular OntoNotes 5.0 scheme, we can use one of the NER algorithms that follows that labeling convention.

To validate the mentions of the Elogld dataset, we decided to use spaCy. SpaCy is one of the most popular libraries for natural language processing, which includes a state-of-the-art NER algorithm. To validate our mentions, we used two NER models. Fast and small: en_core_web_sm and most powerful transformer-based: en_core_web_trf.

**Fig. 13:**
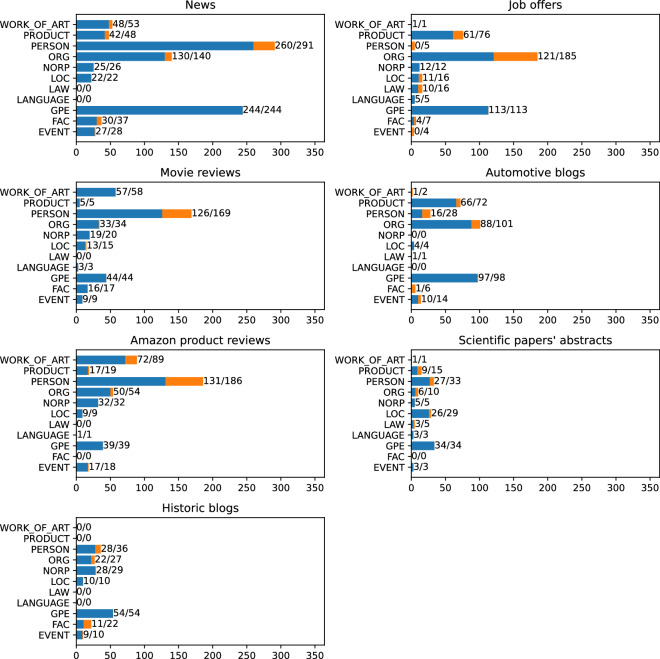
Number of links in each of NER classes after converting to OntoNotes 5.0 scheme, split into separate categories. The first number shows the quantity of linked entities, the second all marked mentions.

Before we could compare the results of spaCy NER with the Elogld annotations, we need to remove the DISEASE, SUBSTANCE, and SPECIE classes from our dataset. This was achieved through Elogld toolset: elgold.py filter --exclude DISEASE --exclude SUBSTANCE --exclude SPECIE. This reduced the total number of annotations to 2803, most importantly shrinking the Scientific papers text category. The distribution of NER classes obtained after filtering is presented in Fig. 13.

**Fig. 14:**
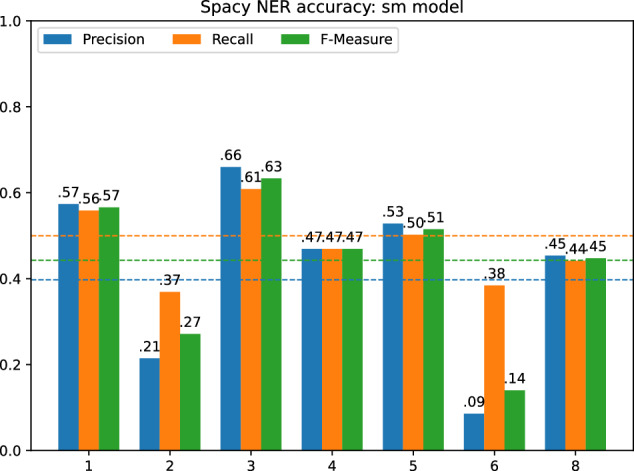
Spacy NER result for different text categories with the average value marked by dashed line, using en_core_web_sm model.

**Fig. 15:**
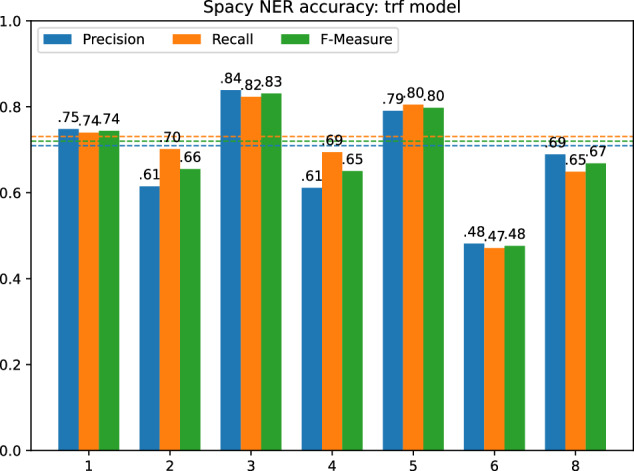
Spacy NER result for different text categories with the average value marked by dashed line, using en_core_web_trf model.

For each NER model, we calculated the precision, recall and the F-measure for the entire dataset and each of the text categories separately. Figure 14 presents the results for en_core_web_sm model, while Fig. 15 presents the results for en_core_web_trf.

For the en_core_web_sm we obtained on average P=0.4, R=0.5, F=0.44. The best results were achieved in News and Movie Reviews, the worst in Job offers and Scientific papers. For the en_core_web_trf the results were much better. We obtained P = 0.71, R = 0.73, and F = 0.72 on average, but the trends in the separate text categories were similar.

Although we can observe that a better model gives better results, which argues for the correctness of our mentions, we still obtain the results below the model’s claimed F-measure = 89.8. We believe that the resulting quality is not due to the low quality of our data but rather to slightly different interpretations of the NER task. Now we will describe some of the most important points of consideration.

**Fig. 16:**
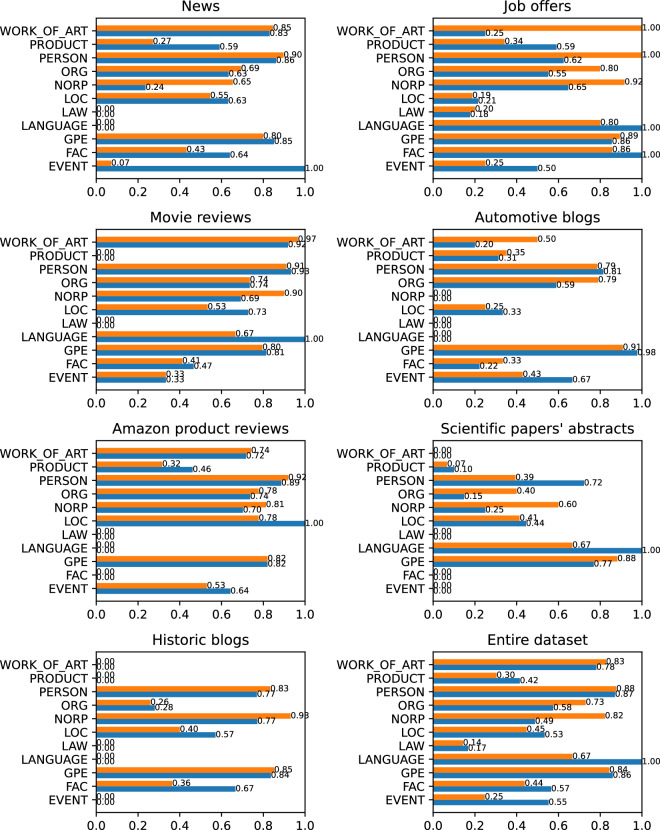
Precision (upper, orange) and recall (bottom, blue) for different text categories and NER classes, using en_core_web_trf model.

First, we must remember that some named entity classes are less precisely defined than others. When we look at spaCy NER results for different classes (Fig. 16), we may observe that GPE, PERSON, WORK_OF_ART were quite easily recognized, while classes like PRODUCT, LOC, or EVENT are much harder. We do not consider the LAW class here since we have too small examples of that class in our Elgold dataset.

When it comes to recognizing the EVENT class, it is very often not that obvious which events should be considered named entities. For example, we would be rather sure that “World War II” should be considered a named entity, but “Russo-Ukrainian War” is not that obvious. The problem with EVENTs (and similarly PRODUCTs) is that the definition of what is and what is not a named entity may change over time. Other classes like PERSON, WORK_OF_ART, or GPE are not so much time dependent.

Another interesting issue comes with the proper classification of noun adjuncts like in phrases: “French President”, “European countries”, or “Zen garden”. The spaCy NER always classifies noun adjuncts like NORP while it is not always true. In the case of country names, it can sometimes refer to a country as a geopolitical unit and sometimes to a nation name. For example, in the case of “French President”, we should rather classify “French” as GPE, as we would do in “President of France”, but in phrases like “French cuisine” we should rather stay with NORP. Similarly in the case of “European countries” we should classify “European” rather as LOC not NORP. In the Elogld dataset, we tried to correctly classify all similar ambiguities that resulted in a lowered accuracy compared to spaCy NER.

Finally, we should also consider that there also exist some minor differences in mention boundaries, like the decision if “the” article should be included in the mention. We include it only when it was part of the official named entity name, for example: “The Times” or “The Royal Ballet”, but “United States” or “Press Association”. SpaCy NER seems to include it more often; however, it is more a matter of style rather than correctness. Another minor difference comes in the decision whether we should include person titles before names like “Mr”, “Mrs”, “Dr”, etc. Spacy NER seems to ignore them all, while we decide to skip the general titles like “Mr” and “Mrs” but keep the more specific ones like “Pope”, “King”, etc. The third common minor issue that exists between the Elogld dataset and spacyNER is the inclusion of the company name before the product model. For example, in our dataset, an entity like “Fiat 2300” is entirely linked to the entity “Fiat 2300”, while spaCy would split into two links. one to the “Fiat” entity and the other to the specific model. It is also hard to say which version is correct. It is more style than correctness.

Considering all these remarks, we believe that the technical validation of our dataset using spacyNER suggests the high quality of detected entities and additionally sheds new light on the problem of Named Entity Recognition, being a valuable source for future research in the field.

### Mention Detection using DBPedia

The mention detection validation with SpaCy does not gives us the information about the quality of named entities from the DISEASE, SUBSTANCE and SPECIE categories. To check the quality for entities in these classes, we used an additional alternative procedure based on a similar technique as that in the section “Measuring texts diversity”.

One again we used the DBPedia Spotlight to annotate raw texts from Elgold dataset, and then mapped the entities categories from DBpedia ontology to Elgold classes (Table 9). Then we can compare the entities found by DBPedia Spotlight with gold-standard entities from Elgold.

We selected 7 random texts from Elgold dataset from each of texts categories. Then for each text, we analyzed the False Positives and False Negatives kinds of errors. The results are presented in Tables 10, 11, 12, 13, 14, 15, 16. For the analyzed sample, we observed that all kinds of differences between the mentions detected by Spotlight and Elgold gold standard are either Spotlight errors or result from the specific assumptions of Elgold dataset. What is an additional indication of Elgold correctness.

**Table 10:** Comparison of DBPedia annotations with Elgold for 1_16.txt.

Sentence	Elgold	DBpedia	C
Leonardo DiCaprio has praised the “amazing work” of an **English** wildlife trust for the success of its osprey breeding programme.	GPE	LANGUAGE	1
The **Titanic** actor is known for his passion for conservation and is a UN climate change representative.	WORK_OF_ART	None	1
The Titanic actor is known for his passion for conservation and is a **UN** climate change representative.	ORG	GPE	1
He posted about the achievements of **Leicestershire and Rutland Wildlife Trust** from his Twitter account.	ORG	None	1
He posted about the achievements of Leicestershire and Rutland Wildlife Trust from his **Twitter** account.	PRODUCT	WORK_OF_ART	1
**DiCaprio** posted the tweet, which included a photo of two ospreys, on Wednesday.	PERSON	None	1
He said it was the first time in nearly two centuries that a pair of osprey chicks had been produced in **England**.	GPE	None	1
However, **Leicestershire and Rutland Wildlife Trust** said the comment was not entirely correct.	ORG	None	1
Mat Carter, from the trust, said: “We are thrilled that Leonardo DiCaprio has posted on social media about the **Osprey Project** at Rutland Water Nature Reserve.	ORG	None	1
Mat Carter, from the trust, said: “We are thrilled that Leonardo DiCaprio has posted on social media about the Osprey Project at **Rutland Water Nature Reserve**.	FAC	None	1
“Our conservation work reintroducing the ospreys back to **England** over the last 25 years, producing 200 chicks is something we are incredibly proud of.”	GPE	None	1
**DiCaprio** - who describes himself as an environmentalist - frequently uses his social media platform to comment on issues related to climate change.	PERSON	None	1
He has co-founded a group called **Earth Alliance** and attended the COP26 summit in Glasgow last year.	ORG	None	1
He has co-founded a group called Earth Alliance and attended the **COP26** summit in Glasgow last year.	EVENT	None	1
Leonardo DiCaprio has praised the “amazing work” of an English **wildlife trust** for the success of its osprey breeding programme.	None	ORG	2
He posted about the achievements of **Leicestershire** and Rutland Wildlife Trust from his Twitter account.	None	GPE	3
He posted about the achievements of Leicestershire and **Rutland** Wildlife Trust from his Twitter account.	None	GPE	3
However, **Leicestershire** and Rutland Wildlife Trust said the comment was not entirely correct.	None	GPE	3
However, Leicestershire and **Rutland** Wildlife Trust said the comment was not entirely correct.	None	GPE	3
Mat Carter, from the trust, said: “We are thrilled that Leonardo DiCaprio has posted on social media about the Osprey Project at **Rutland Water** Nature Reserve.	None	LOC	3

Comment column: 1 – correct entity according to Elgold rules; 2 – not a named entity; 3 – partial match of bigger entity.

**Table 11:** Comparison of DBPedia annotations with Elgold for 2_8.txt.

Sentence	Elgold	DBpedia	C
**IPEX** is one of the North American leading providers of advanced plastic piping systems.	ORG	DISEASE	1
IPEX is one of the **North American** leading providers of advanced plastic piping systems.	LOC	None	1
Principal Responsibilities Verify and update MES (**Hydra**) information as required.	PRODUCT	None	1
Update **Kronos** as the need arises for attendance for reporting team Provide and replace operator?	PRODUCT	WORK_OF_ART	1
Relay new information accordingly Update various reports daily including **Hydra**, Transition Logs, Shift Performance Summary, and Cycle Times.	PRODUCT	None	1
Ensure that finished product is manufactured in compliance with the Corporate Quality Control Manual and **ISO** procedures	ORG	None	1
Experience and familiarity of **ISO 9001** requirements and processes in relation to production an asset	LAW	None	1
Technical understanding of production planning techniques, basic understanding of quality management standards, lean manufacturing and safety requirements/laws (**ISO 9001**, Six Sigma, 5S, etc.)	LAW	None	1
Intermediate **Microsoft Office** skills	PRODUCT	WORK_OF_ART	1
**IPEX** is committed to providing accommodations for people with disabilities throughout the recruitment process and, upon request, will work with qualified job applicants to provide suitable accommodation in a manner that takes into account the applicant?	ORG	DISEASE	1
Accommodation requests are available to candidates taking part in all aspects of the selection process for **IPEX** jobs.	ORG	DISEASE	1
Technical understanding of production planning techniques, basic understanding of quality management standards, lean manufacturing and safety requirements/laws (ISO 9001, Six Sigma, **5S**, etc.	None	PRODUCT	4
Please submit your applications to: https://apply.workable.com/**ipex**/j/A069CF6732/	None	DISEASE	5

Comment column: 1 – correct entity according to Elgold rules; 2 – not a named entity; 3 – partial match of bigger entity; 4 – named entity but outside the set of Elgold classes; 5 – part of URI.

**Table 12:** Comparison of DBPedia annotations with Elgold for 3_2.txt.

Sentence	Elgold	DBpedia	C
The **Empire State Building** is made of rubber.	FAC	None	1
Memo to anyone on the **National Mall**: When the Earth’s crust is shifting, don’t stand within range of the Washington Monument.	FAC	None	1
Memo to anyone on the National Mall: When the **Earth**’s crust is shifting, don’t stand within range of the Washington Monument.	LOC	None	1
Memo to anyone on the National Mall: When the Earth’s crust is shifting, don’t stand within range of the **Washington Monument**.	FAC	None	1
It will come as little surprise (because at this writing the film’s trailer on **YouTube** alone had more than 7,591,413 views) that the aircraft carrier John F.	PRODUCT	WORK_OF_ART	1
It will come as little surprise (because at this writing the film’s trailer on YouTube alone had more than 7,591,413 views) that the aircraft carrier **John F. Kennedy** rides a tsunami onto the White House.	PERSON	None	1
When **St. Peter’s Basilica** is destroyed,	FAC	None	1
**Leonardo**’s God and Adam are split apart just where their fingers touch	PERSON	None	1
Leonardo’s **God and Adam** are split apart just where their fingers touch	WORK_OF_ART	None	1
(the ceiling of the **Sistine Chapel** having been moved into St. Peter’s for the occasion).	FAC	None	1
(the ceiling of the Sistine Chapel having been moved into **St. Peter’s** for the occasion).	FAC	None	1
It will come as little surprise (because at this writing the film’s trailer on YouTube alone had more than 7,591,413 views) that the aircraft carrier John F. **Kennedy** rides a tsunami onto the White House.	None	PERSON	3
**Peter**’s Basilica is destroyed,	None	PERSON	3
(the ceiling of the Sistine Chapel having been moved into St. **Peter**’s for the occasion).	None	PERSON	3

Comment column: 1 – correct entity according to Elgold rules; 2 – not a named entity; 3 – partial match of bigger entity.

**Table 13:** Comparison of DBPedia annotations with Elgold for 4_9.txt.

Sentence	Elgold	DBpedia	C
Nationwide roadside rescue and recovery provider **Start Rescue** has been named the UK’s leading breakdown company.	ORG	None	1
In the annual **Which?** Recommended Provider customer survey,	ORG	None	1
**Start Rescue** came out top for the fourth consecutive year, with the highest percentage of customers feeling they get value for money.	ORG	None	1
The research found that **Start Rescue**’s full home, roadside and national annual cover was more than £94 cheaper than the most expensive equivalent competitor, with its most affordable policy starting at just £19.	ORG	None	1
In taking the top spot in the **Which?** Recommended Provider Breakdown Services Survey	ORG	None	1
Recommended Provider Breakdown Services Survey, **Start Rescue** was placed ahead of some of the biggest names in breakdown, including the AA, RAC and Green Flag.	ORG	None	1
Recommended Provider Breakdown Services Survey, Start Rescue was placed ahead of some of the biggest names in breakdown, including **the AA**, RAC and Green Flag.	ORG	None	1
Recommended Provider Breakdown Services Survey, Start Rescue was placed ahead of some of the biggest names in breakdown, including the AA, RAC and **Green Flag**.	ORG	None	1
**Nationwide** roadside rescue and recovery provider Start Rescue has been named the UK’s leading breakdown company.	None	ORG	2
Recommended Provider **customer survey**, Start Rescue came out top for the fourth consecutive year, with the highest percentage of customers feeling they get value for money.	None	WORK_OF_ART	2

Comment column: 1 – correct entity according to Elgold rules; 2 – not a named entity; 3 – partial match of bigger entity.

**Table 14:** Comparison of DBPedia annotations with Elgold for 5_18.txt.

Sentence	Elgold	DBpedia	C
This review is for the Pink Floyd **London 1966/1967** DVD only, All Regions edition.	WORK_OF_ART	None	1
This promotional film footage shot by director Peter Whitehead of both Interstellar Overdrive and **Nick’s Boogie** (Live/Studio) is quite interesting and of good sound/video quality.	WORK_OF_ART	None	1
Also, I found the interviews with Mick Jagger, Michael Cain, Julie Christie, and David Hockney interesting to varying degrees along with the cool cameo of John Lennon and the **Yoko Ono** Happening scenes.	PERSON	None	1
This review is for the Pink Floyd **London** 1966/1967 DVD only, All Regions edition.	None	GPE	3
This promotional film footage shot by director Peter Whitehead of both Interstellar Overdrive and **Nick**’s Boogie (Live/Studio) is quite interesting and of good sound/video quality.	None	PERSON	3
Also, I found the interviews with Mick Jagger, Michael Cain, Julie Christie, and David Hockney interesting to varying degrees along with the cool cameo of John Lennon and the **Yoko** Ono Happening scenes.	None	PERSON	3
Also, I found the interviews with Mick Jagger, Michael Cain, Julie Christie, and David Hockney interesting to varying degrees along with the cool cameo of John Lennon and the Yoko **Ono** Happening scenes.	None	PERSON	3

Comment column: 1 – correct entity according to Elgold rules; 2 – not a named entity; 3 – partial match of bigger entity.

**Table 15:** Comparison of DBPedia annotations with Elgold for 6b_2.txt.

Sentence	Elgold	DBpedia	C
**Cucullanus tunisiensis** sp. nov.,	SPECIE	None	1
(**Nematoda**: Cucullanidae), collected from the intestine of the white grouper Epinephelus aeneus from waters off the coast of Tunisia is described based on light and scanning electron microscopic observations.	SPECIE	None	1
(Nematoda: Cucullanidae), collected from the intestine of the white grouper **Epinephelus aeneus** from waters off the coast of Tunisia is described based on light and scanning electron microscopic observations.	SPECIE	None	1
This is the sixth nominal species of the genus **Cucullanus Müller, 1777** and the first representative of this genus infecting fishes of Serranidae family reported from Tunisian waters.	SPECIE	None	1
This is the sixth nominal species of the genus Cucullanus Müller, 1777 and the first representative of this genus infecting fishes of **Serranidae** family reported from Tunisian waters.	SPECIE	None	1
This is the sixth nominal species of the genus Cucullanus Müller, 1777 and the first representative of this genus infecting fishes of Serranidae family reported from **Tunisian** waters.	GPE	None	1
**Cucullanus** tunisiensis sp. nov.,	None	SPECIE	3
This is the sixth nominal species of the genus **Cucullanus** Müller, 1777 and the first representative of this genus infecting fishes of Serranidae family reported from Tunisian waters.	None	SPECIE	3

Comment column: 1 – correct entity according to Elgold rules; 2 – not a named entity; 3 – partial match of bigger entity.

**Table 16:** Comparison of DBPedia annotations with Elgold for 8_15.txt.

Sentence	Elgold	DBpedia	C
Exquisitely carved stone murals have been discovered on the east side of the ancient **Zhouqiao Bridge** in Kaifeng in central China’s Henan Province.	FAC	None	1
Exquisitely carved stone murals have been discovered on the east side of the ancient Zhouqiao Bridge in Kaifeng in central **China**’s Henan Province.	GPE	None	1
The murals date to the **Northern Song Dynasty** (960-1127).	ORG	GPE	1
The intricate designs depict auspicious animals - mythical **seahorses** at swim, cranes in flight - and a profusion of small, curly clouds.	SPECIE	None	1
The intricate designs depict auspicious animals - mythical seahorses at swim, **cranes** in flight - and a profusion of small, curly clouds.	SPECIE	None	1
They are the largest **Northern Song Dynasty** stone relief murals ever discovered, and the excavation isn’t even done yet.	ORG	GPE	1
Kaifeng, then called Dongjing, was the capital of the **Northern Song Dynasty**.	ORG	GPE	1
**Zhouqiao Bridge** was built between 780 and 783 A.	FAC	None	1
during the **Tang Dynasty** at the junction of Kaifeng’s main thoroughfare and the Grand Canal, the artificial riverway that connected northern and southern China and is the longest canal in the world.	ORG	GPE	1
during the Tang Dynasty at the junction of Kaifeng’s main thoroughfare and the Grand Canal, the artificial riverway that connected northern and southern **China** and is the longest canal in the world.	GPE	None	1
The bridge was refurbished and expanded in the **Northern Song** period, and the auspicious carvings added.	ORG	GPE	1
Archaeologists believe they will prove to be as much as **100 feet** long when fully excavated.	None	WORK_OF_ART	2
during the Tang Dynasty at the junction of Kaifeng’s main thoroughfare and the Grand Canal, the artificial **riverway** that connected northern and southern China and is the longest canal in the world.	None	FAC	2

Comment column: 1 – correct entity according to Elgold rules; 2 – not a named entity; 3 – partial match of bigger entity.

### Entity Disambiguation

The next stage of our technical validation is the entity disambiguation. In this stage, we take the detected mentions from the dataset, remove their targets, and try to re-create the targets using the selected algorithm. This process is often called linking with gold mentions. This validation stage can tell us if the mentions selected by the annotators can be considered correct.

To validate the Elogld dataset, we selected BLINK library^17^ which is one of the state-of-the-art entity disambiguation systems, which uses Wikipedia as a target KB. Table 17 presents the results of the operation of BLINK in our dataset.

**Table 17:** Blink results.

Dataset	Accepted	Rejected	% rejected	Accuracy
1	781	67	7.90%	0.9316
2	343	7	2.00%	0.9161
3	323	4	1.22%	0.8925
4	270	14	4.93%	0.9317
5	367	2	0.54%	0.8862
6	693	71	9.29%	0.9382
8	157	7	4.27%	0.8377
Entire dataset	2934	172	5.54%	0.9153

The first important thing to notice is the “Rejected” column, which counts the link targets that are unknown to BLINK. All are Wikipedia articles that were created after the 2019/08/01 Wikipedia dump on which the library is based. As we may observe most rejected articles are from the News and Scientific Abstract category. This is reasonable since in the news we usually refer to recent events, and Science also develops dynamically. Overall, we have about 5% rejected targets after 4 years of Wikipedia development.

Another interesting result is the accuracy of the engine for different categories. We may observe that the engine works best on News and Scientific Abstracts but performs surprisingly poorly on Historic blogs. We think that in the case of News, it works best since this is the type of text that most EL engines are optimized for. However, scientific abstracts are quite easy to disambiguate since the mentions are often very specific (line “Nitazoxanide” or “Escherichia coli”). The poor result on Historic Blogs is caused by the problems that BLINK has when some historical geopolitical entities are named the same as their contemporary counterparts (e.g. “Rome” -> “Roman Republic” or “Roman Empire”, “Venice” -> “Republic of Venice”). This again shows that the current EL systems are strongly biased.

The average accuracy of the BLINK on the entire Elogld dataset is 0.91 which is similar to the result obtained by the BLINK authors on the other popular datasets. We consider it a strong indication of the correctness of our dataset.

## Data Availability

The ligilo utility, which was used for text annotation, is released under the MIT license and can be downloaded from GitHub: https://github.com/solewniczak/ligilo. The Elogld toolset, which was used for some automatic verification steps and provides some tools for analyzing and processing the dataset is released under the MIT license and can be downloaded from GitHub: https://github.com/solewniczak/elgold-toolset.
